# Single-Cell Fluorescence Analysis of Pseudotemporal Ordered Cells Provides Protein Expression Dynamics for Neuronal Differentiation

**DOI:** 10.3389/fcell.2019.00087

**Published:** 2019-05-29

**Authors:** Zhichao Song, Alejandra S. Laureano, Kishan Patel, Sylvia Yip, Azadeh Jadali, Kelvin Y. Kwan

**Affiliations:** ^1^Department of Cell Biology and Neuroscience, Rutgers University, Piscataway, NJ, United States; ^2^Stem Cell Research Center and Keck Center for Collaborative Neuroscience, Rutgers University, Piscataway, NJ, United States

**Keywords:** inner ear, stem cell, spiral ganglion neuron, differentiation, regeneration

## Abstract

Stem cell replacement therapy is a potential method for repopulating lost spiral ganglion neurons (SGNs) in the inner ear. Efficacy of cell replacement relies on proper differentiation. Defining the dynamic expression of different transcription factors essential for neuronal differentiation allows us to monitor the progress and determine when the protein functions in differentiating stem cell cultures. Using immortalized multipotent otic progenitors (iMOPs) as a cellular system for SGN differentiation, a method for determining dynamic protein expression from heterogeneous cultures was developed. iMOP-derived neurons were identified and ordered by increasing neurite lengths to create a pseudotime course that reflects the differentiation trajectory. The fluorescence intensities of transcription factors SOX2 and NEUROD1 from individual pseudotemporally ordered cells were measured. Individual cells were grouped by K-means clustering and the mean fluorescence intensity for each cluster determined. Curve fit of the mean fluorescence represented the protein expression dynamics in differentiating cells. The method provides information about protein expression dynamics in differentiating stem cell cultures.

## Introduction

The spiral shaped cochlea harbors the sensory hair cells and spiral ganglion neurons (SGNs) required for hearing. Release of neurotransmitters from hair cells onto the synaptic endings of SGNs initiates transmission of the auditory signal. SGNs are specialized bipolar or pseudounipolar neurons that sustain high rates of neurotransmitter release with high fidelity (Rutherford and Moser, 2016). SGN loss due to loud noise and ototoxins results in sensorineural loss (Kujawa and Liberman, 2006). Exposure to loud sounds in mice show an acute loss of synapses (Kujawa and Liberman, 2015) and delayed degeneration of SGNs (Kujawa and Liberman, 2006, 2009). Cumulative effects from exposure to loud noise significantly contribute to age-related hearing loss (Fernandez et al., 2015). Auditory prosthesis such as hearing aids and cochlear implants require functional SGNs, thus the replacement or regeneration of SGNs is a major milestone for hearing restoration.

Stem cell replacement can alleviate SGN loss if the appropriate differentiation occurs and synapses reformed (Shi and Edge, 2013; Fritzsch et al., 2015; Rutherford and Moser, 2016). Ouabain application to the round window of the cochlea selectively destroys type-I SGNs and is a well-established neuropathy model (Yuan et al., 2014). Using the ouabain neuropathy model, denervated gerbil cochleae were transplanted with human pluripotent stem cell-derived neural otic progenitors (Chen et al., 2012). Engrafted otic progenitors differentiated into neurons, extended neurites to synapse onto hair cells and cochlear nucleus and resulted in partial hearing recovery (Chen et al., 2012). Efficient differentiation promotes functional recovery while improper differentiation of pluripotent stem cells results in teratoma formation and exacerbates an already compromised auditory system (Nishimura et al., 2012). Defining the molecular underpinnings that promote otic progenitor differentiation into SGNs is crucial for successful employment of stem cells replacement therapies.

To study differentiation of otic neural progenitors into nascent neurons, we employed clonally derived immortalized multipotent otic progenitor (iMOP) cells. iMOP cells were generated from SOX2 expressing cochlea progenitors and immortalized by transient expression of C-MYC (Kwan et al., 2015). Under appropriate culture conditions, iMOP cells differentiate into bipolar and pseudounipolar neurons (Jadali et al., 2016). Unlike other neurons that form complex axonal and dendritic branches, iMOP-derived neurons display bipolar or pseudounipolar neurites similar to SGNs *in vivo* (Kiang et al., 1982; Spoendlin and Schrott, 1989; Nayagam et al., 2011). The lack of neurite branching allows straight forward quantification of neurite lengths. Although iMOP cells can differentiate into iMOP-derived neurons, the onset of differentiation is asynchronous. Asynchronous differentiation in iMOP cultures was exploited by acquiring quantitative fluorescent images of cells with different neurite lengths and ordering individual cells based on increasing neurite lengths to generate a pseudo-timeline that represents progression of neuronal differentiation. Quantification of the fluorescence intensity of nuclear proteins in pseudotemporal ordered cells provided insight into protein expression dynamics as cells transitioned from a progenitor into a nascent neuronal state. The method provides insight into protein expression dynamics during neuronal differentiation.

## Results

### Enrichment of Post-mitotic iMOP Cells Using a CDK2 Inhibitor

**FIGURE 1:**
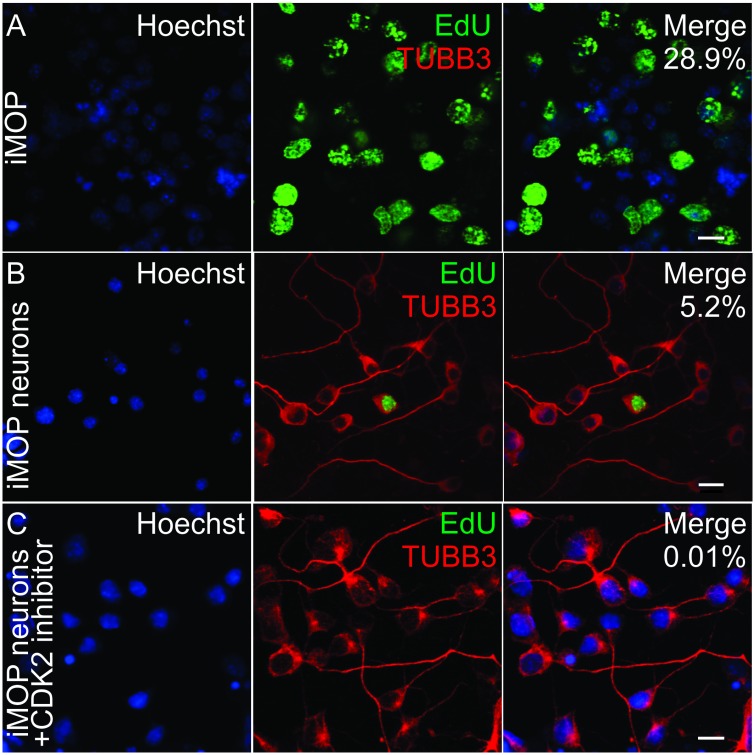
Effects of CDK2 inhibitor in differentiating iMOP cultures. **(A)** Incorporation of the 5-ethynyl-2′-deoxyuridine (EdU) and TUBB3 immunolabeling in proliferating iMOP cells. EdU and TUBB3 labeling in **(B)** iMOP-derived neuron cultures, and **(C)** iMOP-derived neuron cultures treated with 1 μM K03861. Average percentages of EdU marked cells are represented in merged panels (*n* = 3 independent experiments). Scale bars are 10 μm.

Multipotent otic progenitor cells can self-renew as otospheres or differentiate into iMOP-derived neurons when cultured as an adherent culture (Jadali and Kwan, 2016). In iMOP-derived neuronal cultures, cells asynchronously exit the cell cycle to initiate neuronal differentiation. The cyclin dependent kinase 2 (CDK2) in iMOP cells contributes to proliferation (Song et al., 2017). To enrich for post-mitotic cells, a CDK2 inhibitor, K03861 was added to cultures. K03861 competes with cyclin binding to inhibit CDK2 kinase activity and prevent cell cycle progression (Alexander et al., 2015). Concentration of K03861 added to enrich for post-mitotic cells was previously determined using a dose response curve (Song et al., 2017). Cells were cultured under neuronal differentiation conditions in the absence or presence of 1 μM of K03861 before being subjected to 5-ethynyl-2′-deoxyuridine (EdU) incorporation. EdU is a nucleotide analog that incorporates into newly synthesized DNA and serves as an indicator of proliferating cells. To mark differentiating iMOP cells, immunostaining with antibodies against neuronal β-tubulin 3 (TUBB3) was done (Berglund and Ryugo, 1991; Barclay et al., 2011). TUBB3 labeling highlighted neuronal morphology of cells. Cultures from proliferating iMOPs, iMOP-derived neurons cultured in the absence or presence of K03861 were compared. Proliferating iMOP cells showed a robust percentage of EdU labeled cells (29.6%) without TUBB3 labeling (0%) (Figure 1A). In iMOP neuronal cultures, the vast majority of cells were devoid of EdU and labeled with TUBB3 (91.5%). There was a small population of EdU and TUBB3 labeled cells (5.2%) that represent nascent neurons that just exited the cell cycle (Figure 1B). Addition of 1 μM K03861 virtually eliminated EdU labeled cells (0.01%) with the vast majority of cells labeled with TUBB3 (93.8%). Inclusion of K03861 prevented proliferation, enriched for post-mitotic cells in neuronal cultures and allowed cells to undergo neuronal differentiation. In subsequent experiments, all iMOP-derived neuronal cultures contained K03861 (Figure 1C).

### Transcript Levels of Cell Cycle and Neuronal Genes

**FIGURE 2:**
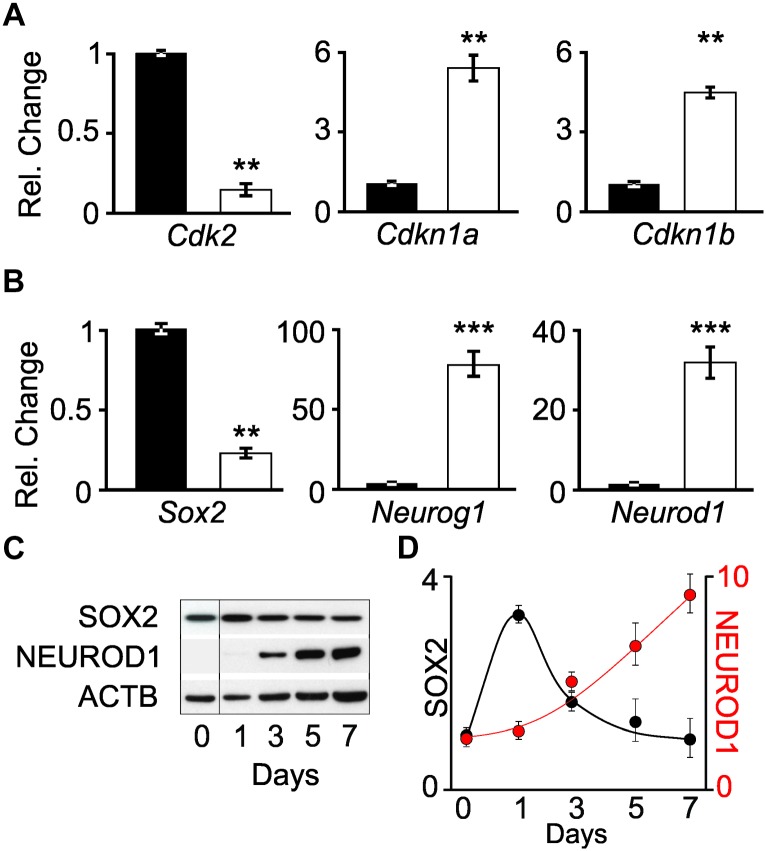
Changes in transcript and protein levels in proliferating iMOP and iMOP-derived neurons. Relative changes in **(A)** cell cycle genes (*Cdk2*, *Cdkn1a*, and *Cdkn1b*) and **(B)** transcription factors (*Sox2*, *Neurog1*, and *Neurod1*) using quantitative PCR (qPCR) in proliferating iMOP (black) and iMOP-derived neurons cultured in the presence of 1 μM K03861 (white) (*n* = 3 independent experiments, ^∗∗^*p* < 1 × 10^-2^ and ^∗∗∗^*p* < 1 × 10^-3^). **(C)** Western blot of SOX2, NEUROD1, and ACTB at different time points during neuronal differentiation. **(D)** SOX2 and NEUROD1 protein levels normalized to ACTB at different time points (*n* = 3 independent experiments).

The differentiation status of cells was determined by measuring the transcript levels of cell cycle genes and transcription factors involved in neuronal differentiation. Quantitative PCR (qPCR) was performed on *Cdk2*, *Cdkn1a* (*p21*^CIP^), and *Cdkn1b* (*p27*^KIP^) transcripts obtained from proliferating iMOP and iMOP-derived neuron cultures. *Cdk2* encodes a cyclin dependent kinase that promotes S phase entry during the cell cycle in iMOP cells (Song et al., 2017). *Cdkn1a* and *Cdkn1b* encode for cyclin dependent kinase inhibitors that bind to CDKs and inhibit cell cycle progression. Normalized *Cdk2* transcript levels from proliferating iMOP cells (1+/−0.03) were significantly decreased compared to iMOP-derived neurons (0.12+/−0.03, *p* < 0.01). Conversely, both cyclin dependent kinase inhibitors *Cdkn1a* and *Cdkn1b* were upregulated. Compared to proliferating cells, *Cdkn1a* (1+/−0.1) significantly increased in iMOP-derived neurons (4.0+/−0.6, *p* < 0.01). A similar increase in *Cdkn1b* was observed when comparing proliferating iMOPs (1+/−0.2) to iMOP-derived neurons (3.3+/−0.8, *p* < 0.01) (Figure 2A). The qPCR results suggest that iMOP-derived neurons showed decreased *Cdk2* concomitant with increased transcript levels of cyclin dependent kinase inhibitors expected for post-mitotic cells.

To determine transcript expression dynamics as the cells transition from a progenitor to a nascent neuron, qPCR was performed on *Sox2*, *Neurog1*, and *Neurod1* transcripts. The aforementioned transcription factors form a transcriptional regulatory network required for progression of inner ear neurogenesis (Evsen et al., 2013). In the developing mouse inner ear, SOX2 progenitors at embryonic day (E) 8.5–11.5 give rise to spiral ganglion neurons (Gu et al., 2016). Conditional knockout of *Sox2* highlights its requirement for SGN specification during otic neurogenesis (Steevens et al., 2017). Similarly, NEUROG1 progenitors are initially expressed in the neural sensory competent domain (NSD) at E9.5 and contribute to spiral and vestibular ganglion (Koundakjian et al., 2007). Ablation of *Neurog1* led to a complete loss of SGNs murine inner ear (Ma et al., 2000). *Neurod1* is a transcription factor required for SGN differentiation and survival (Jahan et al., 2010). The cochlear vestibular ganglion, among other neuronal cell types, are marked by a *Neurod1* reporter (Aprea et al., 2014).

The transcript levels of these genes were employed as indicators of neuronal differentiation. Comparing *Sox2* levels in proliferating iMOPs (1.0+/−0.2) to iMOP-derived neurons cultures (0.34+/−0.006, *p* < 0.01), a decrease in *Sox2* transcripts levels was observed. In contrast, *Neurog1* levels robustly increased during neuronal differentiation when comparing proliferating iMOPs (1.0+/−0.19) to iMOP-derived neurons (104.9+/−13.2, *p* < 0.005). *Neurod1* levels also increased during neuronal differentiation when comparing proliferating iMOPs (1.0+/−0.16) to iMOP-derived neurons (39.4+/−8.9, *p* < 0.005) (Figure 2B). These results suggest a decrease in *Sox2* and increase in *Neurog1* and *Neurod1* transcript levels in iMOP-derived neurons.

### Expression of SOX2 and NEUROD1 Protein During iMOP Neuronal Differentiation

Although comparison of transcript levels in proliferating iMOP to iMOP-derived neuron cultures showed differences, post-transcriptional or translational regulation of the mRNA may not translate into changes in protein levels. Progression of neurogenesis requires inhibition of SOX2 by both NEUROG1 and NEUROD1 (Evsen et al., 2013). To establish that the dynamic expression of SOX2 and NEUROD1 and NEUROG1 quantitative Western blot was done. Since NEUROG1 plays dual roles in both proliferation and neuronal differentiation in iMOP cells (Song et al., 2017), analysis of NEUROG1 was excluded. Lysates from differentiating iMOP cells were collected at different time points during the 7 day differentiation process and used for quantitative Western blotting. SOX2 and NEUROD1 signals were normalized to ACTB (Figure 2C) and compared to levels before differentiation (Day 0). Protein expression was then plotted relative to the number of days under neuronal differentiation (Figure 2D). These results suggest that during iMOP neuronal differentiation, SOX2 levels decrease while NEUROD1 protein levels increased.

Although quantitative Western blots provided bulk protein levels from cultures, the iMOP-derived neuron cultures contain a heterogeneous population of cells at different stages of neuronal differentiation. The averaged protein levels accounts for global changes in the cultures but may mask protein expression from rare cell populations. Single cells can differ dramatically in cellular morphology and protein levels. Variations in protein levels from single cells can be exploited to determine changes protein expression dynamics. By ordering differentiating iMOP cells based on increasing neurite lengths, we would recapitulate the progression of neuronal differentiation. Correlating the protein levels from individual iMOP-derived neurons of different neurite lengths in pseudotemporally ordered cells provides more nuanced temporal dynamics of protein expression.

### Generation of iMOP-Derived Neurons With Heterogeneous Neurite Lengths Using Heterogeneous Cultures

**FIGURE 3:**
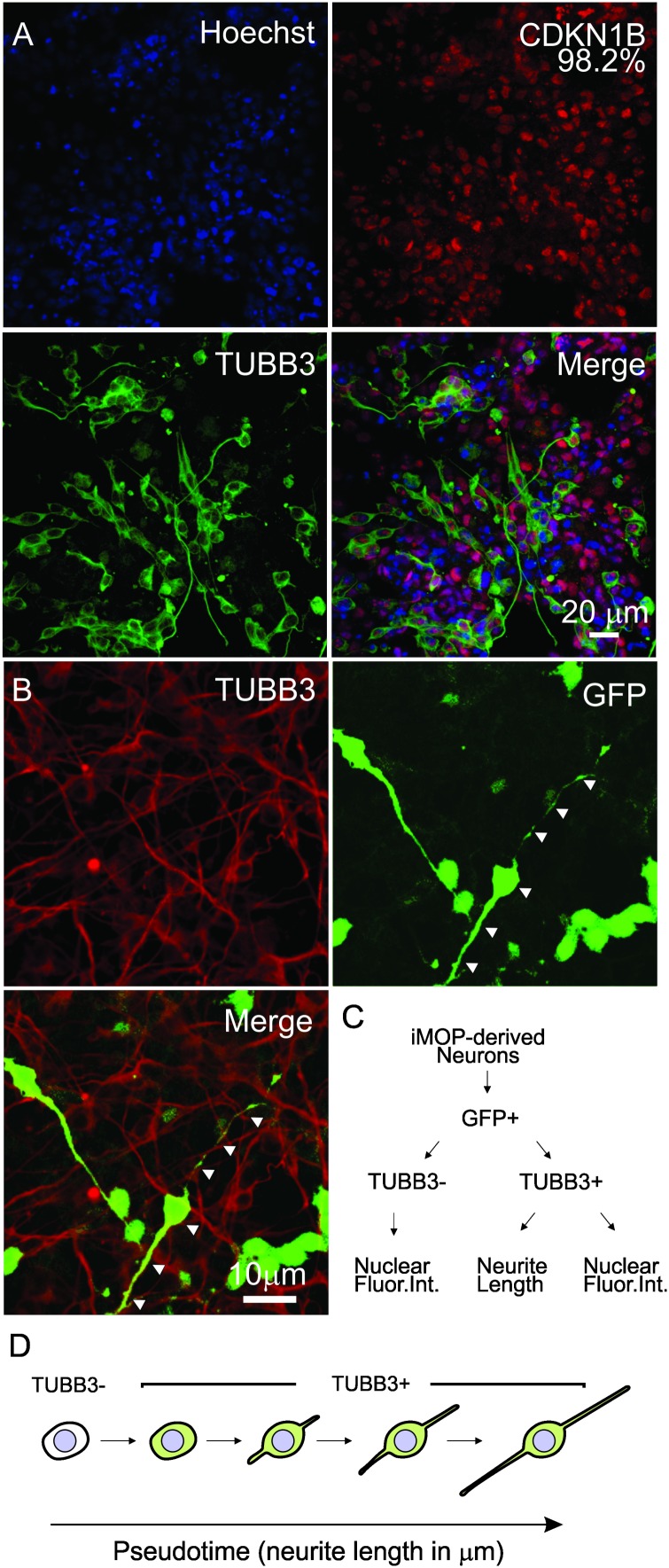
Sparse labeling of iMOP-derived neurons for pseudotemporal ordering. **(A)** Nuclear labeling with Hoechst and immunostaining of iMOP-derived neuron with antibodies against CDKN1B and TUBB3. **(B)** Expression of GFP in TUBB3 marked cells by transfecting a GFP overexpression plasmid for sparse labeling and neurite tracing. Arrowheads mark the path of the neurite in a single neuron. **(C)** Workflow to identify GFP+ TUBB3– and GFP+ TUBB3+ cells for fluoresence intensity and neurite length measurements. **(D)** Arrangement of iMOP-derived neurons based on TUBB3 labeling and neurite lengths to generate a pseudotemporal ordering of cells that represent a differentiation trajectory. Scale bars as noted.

To obtain iMOP-derived neurons that have varying neurite lengths, we obtained early differentiating cells from iMOP-derived neuron cultures 3, 5, and 7 days after initiating differentiation. Immunolabeling showed that essentially all cells expressed CDKN1B (98.2%). Labeling early differentiating iMOP cultures identified both TUBB3+ and TUBB3- cells. TUBB3 labeled cells displayed a wide range of neurite lengths (Figure 3A). To perform automated neurite tracing, unambiguously identification of neurites originating from an individual cell is essential. Sparse culturing of cells decreased survival of iMOP-derived neurons while dense cultures made neurite tracing difficult. Transfecting a small number of cells with a GFP expressing plasmid allows identification and neurite tracing of individual cells (Matsuda and Cepko, 2004). The sparsely labeled GFP expressing cells can be unambiguously identified without overlapping fluorescence signal from labeled cell bodies or neurites. In stark contrast, dense cultures have overlapping labeled cell bodies and neurites that are difficult to visualize and trace. After transfection, a small number of GFP expressing cells in individual fields of view was observed. Colocalization of GFP and TUBB3 suggests that GFP signal alone can be used to label the cell body and trace neurites from individual iMOP-derived neurons even in dense cultures (Figure 3B).

### Pseudotemporal Ordering of Cells With Neurite Lengths

By employing the aforementioned fluorescence labeling paradigm, images were acquired using a high content automated microscope. Images with sparsely labeled GFP cells were identified and GFP cells categorized by the presence or absence of TUBB3 labeling. In TUBB3- cells, the nuclear fluorescence intensities of proteins were measured. In TUBB3+ cells, both the neurite lengths and nuclear fluorescence intensities from individual cells were quantified (Figure 3C). To generate the pseudo-timeline, cells were first categorized based on TUBB3 expression. Cells that did not expressed TUBB3 were placed in the beginning of the pseudo-timeline. Next, TUBB3 expressing cells were ordered according to their neurite lengths. iMOP-derived neurons with neurites up to 250 μm were measured and ordered to form a continuum of cells with increasing neurite lengths (Figure 3D). The pseudotemporal ordered cells represented a differentiation trajectory of post-mitotic iMOP cells undergoing neuronal differentiation. Using the pseudotemporal ordered cells, we quantified relative protein levels in individual cells using quantitative fluorescence microscopy to obtain protein expression dynamics.

**FIGURE 4:**
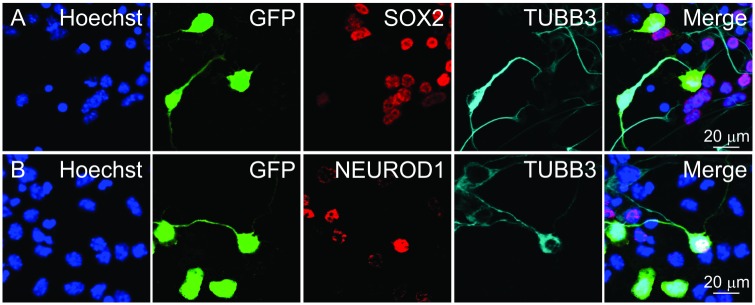
Identifying cells for fluorescence intensity and neurite length measurements. GFP+ cells marked after transfecting a GFP overexpression plasmid. GFP+ cells were categorized based on TUBB3 expression. Individual cells were selected and ordered in pseudotime. Selected cells were then used for measuring nuclear **(A)** SOX2 and **(B)** NEUROD1 fluorescence intensities. Scale bars as noted.

### Establishing Dynamic Expression of Proteins in Pseudotemporal Ordered Cells

To glean information about SOX2 and NEUROD1 expression in differentiating iMOP-derived neurons, nuclear fluorescence intensity for the transcription factors in pseudotemporal ordered cells was done. Differentiating iMOP cultures were transfected with a GFP expressing plasmid to sparsely label individual cells before immunolabeling using antibodies against TUBB3 and either SOX2 or NEUROD1. Fluorescent images containing TUBB3+ or TUBB3- cells were identified. Images of TUBB3 cells were then used to display GFP and SOX2 fluorescence (Figure 4A). Similarly, TUBB3, GFP and NEUROD1 expressing cells were similarly acquired from a separate set of experiments (Figure 4B). GFP labeled cells were identified and neurite lengths measured. The cells were ordered in pseudotime to establish a differentiation trajectory. From these individual cells, the measured fluorescence intensities of SOX2 or NEUROD1 labeling were plotted in pseudotime to obtain protein expression dynamics.

### Clustering of Pseudotemporal Ordered Cells to Correlate Protein Expression With Neurite Lengths

**FIGURE 5:**
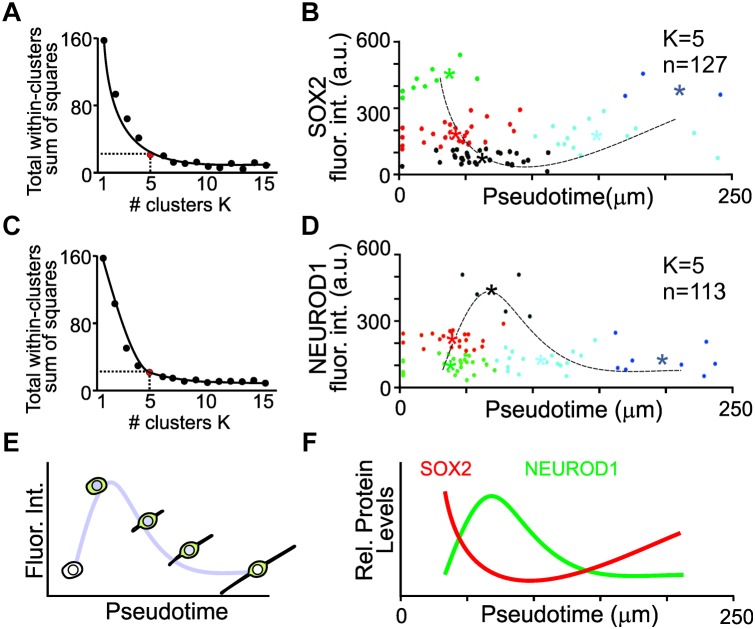
K-means clustering and pseudotemporal analysis of iMOP-derived neurons. **(A)** Elbow test to identify the number of K clusters for SOX2 intensity measurements. Red point denotes the minimal number of clusters that represents the least variance in the data. **(B)** Clustering of cells (*n* = 127) into clusters (*K* = 5). Asterisks denote mean fluorescence intensity of SOX2 for each cluster and the dotted line represents curve fit for the measurements. **(C)** Elbow test for NEUROD1 intensity measurements. **(D)** Clustering of cells (*n* = 113) into distinct populations (*K* = 5). Asterisks represent mean fluorescence intensity of NEUROD1 for each cluster with the curve fit represented by the dotted line. **(E)** Curve fit of the mean fluorescence intensity in pseudotemporal ordered cells represents the dynamics of protein expression. Measurements of cells were obtained from a total of *n* = 5 individual experiments. **(F)** Summary of SOX2 and NEUROD1 protein expression dynamics after pseudotime analysis.

To capture key features from the variable expression levels of individual cells, K-means clustering was used to identify distinct groups of cells that represent changes in protein expression in pseudotime. K-means clustering is a simple unsupervised machine learning algorithm that groups the data into a specified number (K) of clusters (Lloyd, 1982). To determine the minimal number of clusters that represents the least variance in the data, the elbow test was employed (Ketchen and Shook, 1996). The variance for 1–15 clusters was determined. An inflection point of *K* = 5 was identified for the SOX2 expressing cell population (Figure 5A). The fluorescence intensities of SOX2 from pseudotemporal ordered cells were clustered into five different populations and the mean fluorescence intensities from each cluster was determined and used for a simple non-linear least square curve fit to display the expression dynamics of SOX2. SOX2 displayed an initial decrease in expression followed by a slight increase (Figure 5B). Similarly, an inflection point of *K* = 5 was identified for the NEUROD1 expressing cells (Figure 5C). Clustering and curve fitting on the mean fluorescence intensities of NEUROD1 clusters displayed a transient increase in NEUROD1 expression (Figure 5D). By pseudotemporal ordering cells and determining the fluorescence intensity of different transcription factors (Figure 5E), we were able to glean dynamic expression profile of SOX2 and NEUROD1 in differentiating iMOP-derived neurons (Figure 5F). The opposing expression levels of SOX2 and NEUROD1 fit the previously described NEUROD1 negative feedback inhibition on SOX2 during progression of inner ear neurogenesis (Evsen et al., 2013).

## Discussion

In this study, dynamic expression of SOX2 and NEUROD1, transcription factors essential for appropriate otic neuron differentiation were determined. This was achieved by pseudotemporal ordering of cells based on increasing neurite lengths followed by quantitative fluorescence intensity measurements of immunolabeled transcription factors from individual cells. Measurements in individual cells allowed detection of the subtle changes in protein expression relative to neurite lengths. Conventional bulk population analysis of protein such as Western blot masks signals from rare population of cells due to averaging protein expression in an ensemble of cells. The analysis method described provides information about the expression dynamics of proteins.

To accomplish this, we refined the iMOP neuronal cultures by addition of a CDK2 inhibitor, K03861. Addition of K03861 decreased proliferation of iMOP cells, similar to CDK2 siRNA knockdown as shown in our previous study (Song et al., 2017). Addition of K03861 prevented cells from progressing through the cell cycle as evidenced by the decreased numbers of EdU labeled cells and enriched for post-mitotic cells primed for neuronal differentiation. Using the post-mitotic population of cells allowed us to focus on progression of differentiating iMOP cells. Classic *in vivo* experiments using tritiated thymidine or 5-bromo-2′deoxyuridine (BrdU) for birth dating experiments help determine when neurons are born and what neuronal subtypes they become during development (Angevine and Sidman, 1961; Taupin, 2007). These methods for studying neurogenesis are only possible when the anatomy and histological location of a cell is known. *In vitro* stem cell cultures systems lack anatomical landmarks that provide information about cellular differentiation. Instead pseudotemporal ordering in the iMOP culture system provides a differentiation trajectory.

Exploiting the heterogeneity of iMOP-derived neurons, cells with different neurite lengths from cultures were identified. Although there were many iMOP-derived neurons, the intricate and overlapping neurites made neurite tracing difficult. Similar to the Golgi method, iMOP-derived neurons were sparsely labeled by transfecting cells with a GFP expressing plasmid. Expression of GFP in individually identifiable cells allowed for automated detection and neurite length measurement in an otherwise dense culture where neurites are difficult to unambiguously trace. By arranging iMOP-derived neurons based on neurite lengths, the continuum of cells represented the differentiation trajectory in a pseudotime course. Measurement of the fluorescence intensity in cells that represent the differentiation trajectory provides dynamic expression of the proteins. Understanding protein expression dynamics provide insight into their requirement at different stages of differentiation and allows us to determine the extent of neuronal differentiation.

## Materials and Methods

### Cell Culture for iMOP Cells

Multipotent otic progenitor cells were grown in suspension with DMEM/F12 (Life Technologies) containing B27 supplement (Life Technologies), 25 μg/ml carbenicillin and 20 ng/ml bFGF (PeproTech). Cells were cultured in a 96 well flat bottom μclear plate (Greiner) or a 1.5 coverglass (Electron Microscopy Sciences) for high content microscopy. Wells or coverglass were coated with 10 μg/mL of poly-D-lysine for 1 h aspirated and allowed to dry for 15 min, coated overnight with 10 μg/mL of laminin at 37°C, washed 3 times with PBS before plating cells. For neuronal cultures, cells were dissociated in HBSS containing 1 mM EDTA and diluted in neurobasal media before counted using a Moxi cell counter (Orflo). 1.5–2 × 10^4^ cells were seeded in a single poly-D-lysine and laminin coated 96 well and cultured in neurobasal media (Life Technologies) containing B27 and 2 mM L-glutamine (Life Technologies) and 1 μM K03861 at the time of plating. Medium was changed every other day. Cells were maintained in medium for up to 7 days before used for immunostaining.

### Transfection of iMOP-Derived Neurons

To genetically mark iMOP cells with GFP, iMOP cells were seeded for neuronal differentiation and allowed to recover for 24 h before transfection. A pCAG-GFP plasmid was transfected into cells using jetPRIME (Polyplus) using a ratio of 1 μg DNA to 3 μl of JetPRIME transfection reagent. pCAG-GFP was a gift from Connie Cepko (Addgene plasmid # 11150; RRID:Addgene_11150)^1^. The transfection mix containing plasmid DNA, transfection buffer, and transfection reagent was prepared according to the manufacturer. Transfection mix was added to the differentiating cultures, removed from the cultures after 4–6 h and replaced with fresh medium. Cells were allowed to undergo differentiation for another 2 days before immunostaining. The decreased time amount of time incubating the transfection mix with the cultures decreased transfection efficiency and allowed GFP expression in a sparse number of cells that showed no overlapping cell bodies or neurites.

### mRNA Expression Analysis

Total RNA was extracted using Trizol reagent (Life Technologies) and 1 μg of total RNA was used to make cDNA using the qScript cDNA synthesis kit (Quanta Biosciences) according to manufacturer instructions. Relative levels of cDNA were measured by quantitative real time PCR (qPCR) using SYBR green qPCR mix (Life Technologies) for 40 cycles of 95°C for 15 s, 60°C for 1 min using the StepOnePlus real-time PCR machine (Life Technologies). Three biological replicates, each with technical triplicates, were used for each qPCR sample. Samples were normalized to *Actb* and compared to proliferating iMOP samples using the ΔΔC_T_ method. Primers used for qPCR are listed in Table 1.

**Table 1:** Primers for qPCR.

Name	Gene	Sequence 5′- > 3′
*Cdk2* F	*Cdk2*	CCTGCTTATCAATGCAGAGGG
*Cdk2* R	*Cdk2*	TGCGGGTCACCATTTCAGC
*Cdkn1a* F	*Cdkn1a*	CCTGGTGATGTCCGACCTG
*Cdkn1a* R	*Cdkn1a*	CCATGAGCGCATCGCAATC
*Cdkn1b* F	*Cdkn1b*	TCAAACGTGAGAGTGTCTAACG
*Cdkn1b* R	*Cdkn1b*	CCGGGCCGAAGAGATTTCTG
*Sox2* F	*Sox2*	GCGGAGTGGAAACTTTTGTCC
*Sox2* R	*Sox2*	CGGGAAGCGTGTACTTATCCTT
*Neurog1* F	*Neurog1*	CCAGCGACACTGAGTCCTG
*Neurog1* R	*Neurog1*	CGGGCCATAGGTGAAGTCTT
*Neurod1* F	*Neurod1*	ATGACCAAATCATACAGCGAGAG
*Neurod1* R	*Neurod1*	TCTGCCTCGTGTTCCTCGT
*Actb* F	*Actb*	GGCTGTATTCCCCTCCATCG
*Actb* R	*Actb*	CCAGTTGGTAACAATGCCATGT

### Western Blot Analysis

For quantitative Western blots, cells were placed in lysis buffer (50 mM Tris/HCl, pH 7.5, 150 mM NaCl, 1 mM EDTA, 1 mM EGTA, 1% Triton X-100, and 10% glycerol) containing phosphatase inhibitor (Thermo Fisher Scientific) and a mixture of protease inhibitors (Roche). Protein lysates (30 μg) were loaded and separated on 4–12% Bis Tris Novax NuPAGE gradient gels (Life Technologies), transferred to nitrocellulose membrane (GE Healthcare). The membrane was dried overnight and incubated in blocking buffer (1× PBS containing 5% non-fat dried milk) for 1 h, followed by incubation with primary antibodies against SOX2, NEUROD1, and ACTB. Primary antibodies were detected using IRDye 800CW or IRDye 680RD conjugated secondary antibodies (1:10,000 dilution) (LI-COR Biosciences). Fluorescence from the membrane was acquired using the Odyssey imaging system (LI-COR Biosciences) and quantified using the Image Studio software (LI-COR Biosciences). Primary antibodies for quantitative Western blotting are listed on Table 2.

### Immunofluorescence Labeling and Acquisition of Fluorescent Micrographs

Multipotent otic progenitor cells were fixed in 4% formaldehyde in 1× PBS for 30 min, permeabilized in wash buffer (PBS and 0.1% Triton X-100) for 10 min, incubated in blocking buffer (PBS, 10% goat serum and 0.1% Triton X-100) for 1 h and incubated overnight with primary antibody in blocking buffer. Cells were incubated with primary antibodies against SOX2, NEUROD1, CDKN1B, or TUBB3. After incubation with primary antibodies, cells were rinsed in wash buffer and incubated with appropriate combinations of Hoechst (100 ng/ml), Alexa Fluor 488 (1:5,000 dilution), Alexa Fluor 568 (1:5,000 dilution), or Alexa Fluor 647 (1:5,000 dilution) conjugated secondary antibodies (Life Technologies) in blocking buffer for 2 h. Primary antibodies for immunostaining are listed in Table 2.

**Table 2:** Antibodies for Western blot or immunostaining.

Antibody	Company	Catalog #	Dilution for Western or Immunostaining
SOX2	EMD Millipore	ab5603	1:1000
NEUROD1	AbCam	ab15580	1:1000
CDKN1B	Active Motif	39159	1:500
TUBB3	BioLegend	801202	1:1000
ACTB	Santa Cruz Biotechnology	sc1615	1:2000

Quantitative fluorescence images were acquired using a 40× 1.0 NA air objective using the In Cell Analyzer 6000 (GE Healthcare), a high content microscope, and saved as 16 bit images. Background signal was established as fluorescence from cells labeled with only secondary antibodies. To identify transfected cells expressing GFP, nuclear masks were expanded to identify the cell body using a collar algorithm. Individual GFP expressing cells were identified in single fields of view and subjected to neurite tracing using the InCell Analysis software. The subsequent data generated neurite lengths from single cells that range from 0 to 250 μm in length. To confirm software tracing of neurite lengths were accurate, the same images were subjected to manual tracing using Neurite Tracer in ImageJ. To quantify nuclear fluorescence intensity of different proteins, Hoechst labeled nuclei were used to define and generate a mask. The nuclear masks were then used to determine the nuclear fluorescence intensity of SOX2 and NEUROD1.

### Pseudotime Analysis and K-Means Clustering

For each labeled cohort of cultures, 110–130 individual GFP marked cells were identified from 3 independent experiments and analyzed. In GFP labeled cells that did not express TUBB3, the nucleus was identified by Hoechst labeling. In GFP and TUBB3 labeled cells, neurite lengths were measured from bipolar and pseudounipolar iMOP-derived neurons. Only iMOP-derived neurons that had neurites within a single field of view were used for analysis. For the datasets, cells with neurite lengths from 0 to 250 μm and 0–6,000 fluoresence arbitrary units were used. Since fluorescence intensity and the neurite lengths from cells are in different units, display different ranges and not directly comparable, the data points were standardized (Mohamad and Usman, 2013). Fluorescence intensity and neurite lengths were rescaled to have a mean value of 0 and standard deviation of 1 before performing K-means clustering. After K-means clustering the values were converted back to the appropriate scale. To determine the optimal number of clusters in the dataset, the elbow test was employed. The elbow test was performed to determine the sum of squared errors for 1–15 clusters. A minimal number of 5 clusters for SOX2 and NEUROD1 labeled cells achieved a reduction in sums of squares of over 80% was used for K-means analysis. Individual cells were color coded according to their clusters and centroids for each cluster was denoted by an asterisk. Curve fit was done using the centroids. All analysis was performed using R.

### Statistical Analysis

For all experiments, three independent experiments were done. The results from independent experiments were measured and averaged to obtain the presented values. Technical triplicates were included in each experiment to ensure consistency. All error bars shown in data are expressed as +/− standard error (se) of values obtained from independent experiments unless otherwise stated. An unpaired two-tailed Student’s *t*-test was used to determine statistical significance and associated with the appropriate *p*-value. For all figures *p*-values were defined as: ^∗^*p* < 0.05, ^∗∗^*p* < 1 × 10^−2^ and ^∗∗∗^*p* < 1 × 10^−3^ unless otherwise stated.

## Author Contributions

ZS and AL initiated the project, cultured iMOP cells, performed immunostaining, acquired and analyzed the data. KP performed qPCR and immunostaining. SY and ZS performed K-means clustering and curve fitting. AJ performed immunostaining and Western blotting. KK conceived the project, performed the analysis, and wrote the manuscript.

## Conflict of Interest Statement

The authors declare that the research was conducted in the absence of any commercial or financial relationships that could be construed as a potential conflict of interest.

## Footnotes

1 http://n2t.net/addgene:11150
